# Systematic Review of the Properties of Tools Used to Measure Outcomes in Anxiety Intervention Studies for Children with Autism Spectrum Disorders

**DOI:** 10.1371/journal.pone.0085268

**Published:** 2014-01-21

**Authors:** Sarah Wigham, Helen McConachie

**Affiliations:** Institute of Health & Society, Newcastle University, Newcastle, United Kingdom; Birkbeck, University of London, United Kingdom

## Abstract

**Background:**

Evidence about relevant outcomes is required in the evaluation of clinical interventions for children with autism spectrum disorders (ASD). However, to date, the variety of outcome measurement tools being used, and lack of knowledge about the measurement properties of some, compromise conclusions regarding the most effective interventions.

**Objectives:**

This two-stage systematic review aimed to identify the tools used in studies evaluating interventions for anxiety for high-functioning children with ASD in middle childhood, and then to evaluate the tools for their appropriateness and measurement properties.

**Methods:**

Electronic databases including Medline, PsychInfo, Embase, and the Cochrane database and registers were searched for anxiety intervention studies for children with ASD in middle childhood. Articles examining the measurement properties of the tools used were then searched for using a methodological filter in PubMed, and the quality of the papers evaluated using the COSMIN checklist.

**Results:**

Ten intervention studies were identified in which six tools measuring anxiety and one of overall symptom change were used as primary outcomes. One further tool was included as it is recommended for standard use in UK children's mental health services. Sixty three articles on the properties of the tools were evaluated for the quality of evidence, and the quality of the measurement properties of each tool was summarised.

**Conclusions:**

Overall three questionnaires were found robust in their measurement properties, the Spence Children's Anxiety Scale, its revised version – the Revised Children's Anxiety and Depression Scale, and also the Screen for Child Anxiety Related Emotional Disorders. Crucially the articles on measurement properties provided almost no evidence on responsiveness to change, nor on the validity of use of the tools for evaluation of interventions for children with ASD.

**PROSPERO Registration number:**

CRD42012002684.

## Introduction

The choice of relevant outcomes, and of robust tools to measure those, is a vital stage in the design of evaluation of clinical interventions for children. Where tools are reliable and valid, and outcomes important to children and families, the findings can inform parents, clinicians, researchers, service providers and policy makers about which interventions are most effective. However, to date the outcome measures used for intervention trials for children with autism spectrum disorder (ASD) are too varied to allow sensible decisions about what interventions might be most effective [1]; [2].

Meta-analyses can increase the power of findings by pooling data from individual studies. For example, a meta-analysis of the Revised Children's Manifest Anxiety Scale across 43 studies has found evidence of validity and responsiveness to treatment [3]. Cross-study syntheses of outcome evidence such as this are much needed in the field of ASD, because individual trials are in the main very small and include broad age groups [4]; [5]. There have been discussions of these problems and suggestions of which outcome measures to use [6]; [7], but no widespread uptake in ASD studies.

The focus of the current review is on how to choose appropriate and robust tools to measure outcomes of interventions for a common problem encountered by high-functioning children with ASD – how to cope with symptoms of anxiety in the period of middle childhood. With around 40 per cent having symptoms at the severity of an anxiety disorder [8], and the prevalence of ASD being around 1 per cent [9], this is an important public health problem. In the UK, a government initiative titled ‘Increasing Access to Psychological Therapies’ (IAPT) [10] has since 2012 been extended to children's mental health services, with cognitive behaviour therapy (CBT) for problems such as anxiety and depression as one of the core strands. Outcome monitoring is embedded in the programme.

It is important to have a choice of reliable measurement tools for a particular health condition in order to capture relevant outcomes, and different points of view including patient reported outcomes [11]. A choice of measurement tools also facilitates answering a range of research questions, tailored to the objectives of the intervention, ideally meeting the needs of particular developmental stages [12], and allowing different tools to be used for study outcome evaluation and for selection criteria [13]. Without choice of appropriate tools the benefits of an intervention may be missed or inflated [11]; [14].

In this systematic review, the tools used to measure outcomes in evaluations of clinical interventions for anxiety in children with high-functioning ASD in middle childhood are identified and their quality assessed. Middle childhood is defined here as 8 to 14 years of age during which time children will be entering puberty, beginning some level of personal independence from their parents, and experiencing transition between primary and secondary school. We focus on high-functioning ASD as the children are likely to be able to participate in verbally-loaded interventions such as CBT, even although the prevalence of comorbid psychiatric conditions is similar across IQ and levels of adaptive behaviour [15]. This systematic review will facilitate recommendations of robust tools for use in anxiety intervention trials for children with high-functioning ASD in middle childhood.

The review was conducted in two stages. In stage 1, identification of tools was done by systematic search for literature describing studies of treatment interventions for anxiety in ASD in middle childhood. Then in stage 2, searches focused on the tools used to measure primary outcomes, and articles about these tools were examined for evidence of appropriateness and measurement properties.

## Review Methods: Stage 1

The review protocol was registered online with the International Prospective Register of Systematic Reviews (Registration number: CRD42012002684) and can be accessed at (http://www.crd.york.ac.uk/PROSPERO/prospero.asp). The protocol also pertains to social skills interventions, though only the anxiety interventions and outcome tools are reported here. Preferred Reporting Items for Systematic Reviews and Meta-Analyses (PRISMA) standards are followed in this report (see Checklist S1).

### Search Strategy

The following electronic bibliographic databases were searched: MEDLINE, EMBASE, ERIC, PsycINFO, The Cochrane Library (Cochrane Database of Systematic Reviews, Cochrane Central Register of Controlled Trials (CENTRAL), and Cochrane Methodology Register). The search strategy included the terms shown in Table 1 which were combined using database-specific filters, where these were available. The search was restricted to articles in English, and those published between 1992 and February 2013, the date when the last searches were run. The term Asperger Syndrome was first included as a separate diagnosis in the WHO International Classification of Diseases in 1992 [16] so we expected separate identification of groups of children with ability in the average range to be more frequent and consistent in studies after this date.

**Table 1:** Stage 1 review search terms.

1. (ASC or ASD or Asperg$ or Autis$ or high functioning or communicat$ or Kanner$ or language delay$ or pervasive developmental disorder$ or language disorder$ or HFA or autistic disorder or child development disorders).tiab
2. (child$ or school$ or pediatric$ or paediatric$ or special needs or teenage$ or adolescent$ or youth$).tiab
3. (behavio$ or intervent$ or program$ or rehabilit$ or therap$ or train$ or treat$ or verbal or cognitive behavio$ therapy or CBT or pervasive therapy or outcome assessment or treatment outcome).tiab
4. (worr$ or stress$ or phobi$ or anxiety or phobic disorders).tiab
5. randomi#ed controlled trial.tiab
6. random$.tiab
7. comparative stud$.tw
8. prospective stud$.tw
9. treatment effectiveness evaluation/
10. intervention$.tiab
11. 5 or 6 or 7 or 8 or 9 or 10
12. 1 and 2 and 3 and 4 and 11
13. limit 12 to English language; 1992 – current; age 8 to 14 years

### Selection Criteria

Anxiety was clinically defined as in the International Classification of Diseases [16] and the Diagnostic and Statistical Manual of Mental Disorders [17]. The interventions included cognitive and behavioural approaches, and excluded drug trials, physiological interventions (e.g. biofeedback) and purely physical interventions (e.g. massage). Intervention studies where a broad range of skills were the target (e.g. social skills, or drama classes) were excluded. The interventions included were ameliorative, preventative or educational, aimed at managing and regulating emotional reactions which may be precursors to anxiety disorder.

Studies were included when over 50% of participants were aged 8 to 14 years old, or the mean age of the ASD sample was within this range, so that measures were likely to be appropriate across the target age range for the review. Where child participants had a range of differing diagnoses, the study was included if ASD outcome data were presented separately, and if half or more of participants have ASD.

Group studies with designs including before-and-after, controlled trials, quasi-experimental, and randomised controlled trials (RCT) were included. Studies which used only observational methods of recording outcomes (e.g. event recording) were excluded. The review was restricted to articles published in English.

One reviewer (SW) screened the titles and abstracts of articles; where there was doubt whether an article met the inclusion criteria it was included. Full text sifting was by one reviewer (SW); any ambiguous papers were discussed with the second reviewer (HM) to reach consensus. The references of the selected articles were searched.

### Data extraction

Data extraction was performed by one reviewer (SW) using a previously tested data extraction form. The following information was noted: participant characteristics, focus of intervention, outcome tools used, domains captured, and by whom the tool was reported/measured.

## Results: Stage 1

**Figure 1:**
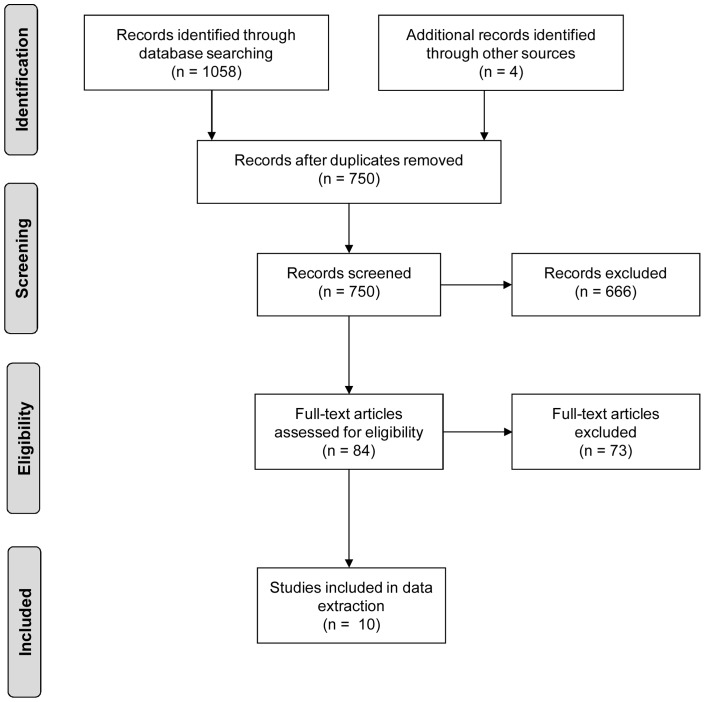
Search strategy flow diagram at Stage 1.

The searches retrieved 750 articles from which 10 articles were retained [18]–[27]. See Figure 1 for search strategy flow diagram. Nine articles report on seven RCTs of adapted CBT for anxiety delivered to high-functioning children with ASD in middle childhood. These studies varied in sample size from 22 to 71 participants, used varied approaches and materials, and included from 6 group sessions [26] to 16 group [23] or individual sessions [19]–[21]. The before-and-after study included 6 participants in 16 group sessions of CBT [25]. All but two [23]; [25] included training for parents. Taken together the studies provide encouraging evidence that CBT can be efficacious for children with ASD and anxiety disorder.

Seven different primary outcome tools were used in these studies. The Anxiety Disorders Interview Schedule (ADIS) [28] is a clinician-administered interview. Five are parent and self-report child anxiety questionnaires [29]–[33]. One further tool is a clinician or researcher rating of overall improvement, the Clinical Global Impressions – Improvement (CGI-I) [34]. No intervention studies meeting our inclusion criteria used the Revised Children's Anxiety and Depression Scale (RCADS) [35]; however as it is an IAPT recommended outcome tool it was also included in stage 2 (Table 2). None of the tools was developed specifically for children with ASD. All of the tools were developed in English (though at stage 2 some articles evaluating the measurement properties of the SCARED were on revised versions developed in Dutch).

**Table 2:** Characteristics of the tools used in included studies.

Measure	Version	Year published	Aim of tool	Number of items	Subscales	Response options	Format	Used in references
**Anxiety Disorders Interview Schedule (ADIS)**	C	1996	Measure of anxiety and related disorders.	(depends on entry questions)	Separation anxiety; social phobia; specific phobia; panic disorder; agoraphobia; generalised anxiety; obsessive-compulsive; post-traumatic stress disorder	9 point severity scale	Semi structured diagnostic interview	19, 20
	P							18, 19, 20, 21, 22
**Multidimensional Anxiety Scale for Children (MASC)**	C + P	1997	Measure of child anxiety	39	Physical symptoms; harm avoidance; social anxiety; separation anxiety.	4 point scale	Questionnaire	19
**Revised Children**'**s Anxiety and Depression Scale (RCADS)**	C	2000	Measure of child anxiety and depression	47	Separation anxiety; social phobia; generalized anxiety; panic; obsessive-compulsive; major depressive disorder.	4 point scale	Questionnaire	**
	P	2010						
	Short	2012		25	Anxiety; depression			
**Revised Children**'**s Manifest Anxiety Scale (RCMAS)**	C	1978	Measure of child anxiety	37	Physiological anxiety; worry/over-sensitivity; fear/concentration; lie scale (social desirability).	Yes/No	Questionnaire	24
	P	2000						
**Screen for Child Anxiety Related Emotional Disorders (SCARED)**	C + P	1997	Measure of child anxiety disorders	38	Panic; generalized anxiety; separation anxiety; social phobia; school phobia.	3 point scale	Questionnaire	18, 27
		1999		41				
	*71 C + P	2009		71	Panic; generalized anxiety; social phobia; separation anxiety; obsessive-compulsive; post-traumatic stress; specific phobias (animal; blood/injury; situational).			
	*Revised C	1999		66				
**Social Worries Questionnaire (SWQ)**	C	1995	Behavioural indicators of social anxiety in children	13	none	3 point scale	Questionnaire	26
	P			10				
**Spence Children**'**s Anxiety Scale (SCAS)**	C	1998	Measure of child anxiety disorders	44	Panic/agoraphobia; separation anxiety; social phobia; obsessive-compulsive; physical injury fears; generalized anxiety disorder.	4 point scale	Questionnaire	21, 22, 23, 24, 25, 26
	P	2004		38				21, 22, 24, 25, 26
**Clinical Global Impressions (CGI)**	Improvement	1976	Measure of clinical improvement	1	none	8 point scale	Clinical rating	18, 19, 22 (23)***

C =  child self report; P =  parent report; NA =  not applicable.

developed in Dutch (all others English). ** not used in intervention evaluation studies reviewed. *** used Clinical Global Impressions-Severity ratings.

## Review Methods: Stage 2

In order to assess the measurement properties of the tools, a comprehensive search was conducted using a methodological search filter designed to locate articles on measurement properties in PubMed [36].

### Data extraction method

Once identified, the methodological quality of each article was examined using the COSMIN checklist (COnsensus based Standards for the selection of health based Measurement INstruments). The checklist considers 9 properties of measurement, each with multiple items rated on a 4 point scale: internal consistency, reliability, measurement error, content validity, structural validity, hypothesis testing, criterion validity, responsiveness to change (and cross-cultural validity, not considered in the present review). For each article, the properties addressed are given an overall rating of excellent, good, fair, poor based on the lowest item rating awarded [37]. The checklists were completed by one reviewer (SW) with frequent discussion of ratings with a second reviewer (HM) to reach consensus. To check reliability the second reviewer independently rated 10% of the articles using the checklist. Agreement on final rating of each property was 71.5%.

### Evidence Synthesis

The quantitative findings in each study were then given a quality rating of positive, indeterminate or negative for each measurement property examined [38]. For example, internal consistency is considered positive where Cronbach's alpha is equal to or greater than 0.70; criterion validity is considered positive where there are convincing arguments that the gold standard is ‘gold’ and correlation is equal to or greater than 0.70.

Finally the quality ratings for the findings were considered in conjunction with the quality rating for the level of evidence in the articles about each tool [38]. This synthesis records *strong* evidence (+++ or −−−) where several methodologically good articles, or one excellent article, find consistent evidence for or against a measurement property; *moderate* evidence (++ or −−) for several methodologically fair, or one good study; a rating of *limited* (+ or −) for one study of fair quality; and otherwise a rating of *conflicting* evidence (+/−) or *unknown* (?) evidence [38].

## Results: Stage 2

**Figure 2:**
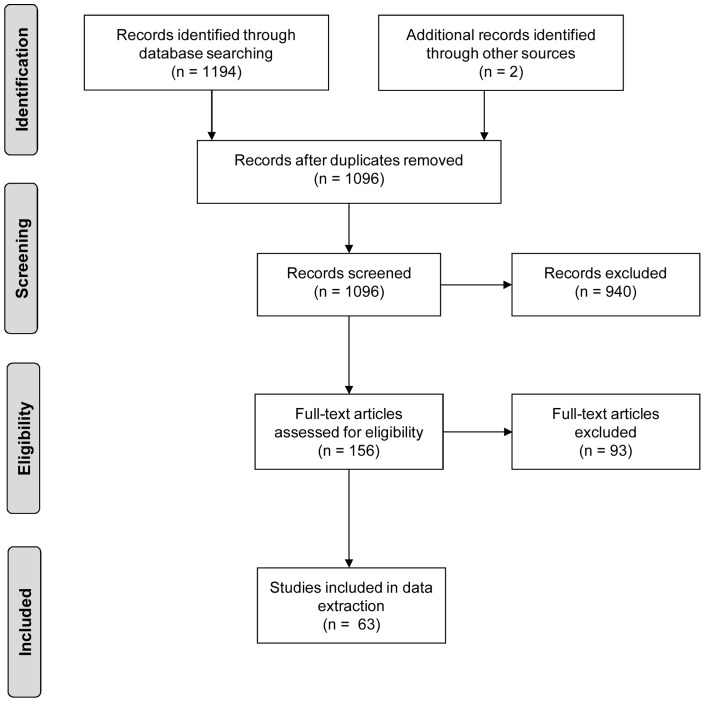
Search strategy flow diagram at Stage 2.

The search in PubMed produced 1096 articles from which 63 were retained for data extraction (Figure 2). The study population characteristics for these articles are shown in Table 3.

**Table 3:** Characteristics of study populations in articles on measurement properties.

Measure	Article	Ref No.	Study Population/sample		Mean age (SD) years; range	N	Male % (child)	Country
**ADIS**	Brown-Jacobsen et al (2011)	40	Anxiety diagnostic clinic		12.32(3.3); 7–18	88	55.7	USA
	Canavera et al (2009)	41	OCD		12.17 (X); 6–17	93	46.2	USA
	Comer & Kendal (2004)	42	Anxiety disorder clinic		10.2 (1.8); 7.5–14	98	54	USA
	Grills & Ollendick (2003)	43	Psychology clinic		10.69 (2.66); X	165	63.6	USA
	Higa-McMillan (2008)	44	CBT centre		12.3 (3.3); 6–18.2	289	68.2	Hawaii
	Lyneham et al (2007)	45	Anxiety disorder clinic		11.25 (2.58); 7–16	153	50.3	Australia
	Lyneham & Rapee (2005)	46	Anxiety clinic and community		9.2 (1.9); 6–12	73	67	Australia
	Silverman et al (2001)	47	Psychosocial research centre		10.15 (X); 7–16	62	39.6	USA
	Storch et al (2012)	48	ASD		10.34 (2.21); 7–17	85	76.5	USA
	Wood et al (2002)	49	Anxiety disorder clinic		11.71 (2.64); 8–17	186	53.8	USA
**MASC**	Anderson et al (2009)	50	School		14.5 (X); 13–17	372	45.7	USA
	Baldwin & Dadds (2007)	51	Community		11.36 (1.21); 9.16–14.42	452	46	Australia
	Brown et al (2012)*	52	Paediatric primary care clinic		12.3 (2.7); 7–17	229	36.6	USA
	Dierker et al (2001)	53	School		14 (X); 13–15	632	45	USA
	Grills-Taquechel et al (2008)	54	Mixed clinical diagnoses		10.44 (2.64); 7–17	262	65	USA
	Langer et al (2010)	55	Anxiety clinic		11.61 (2.64); 6–17	174	54	USA
	March et al (1997)	56	Clinical		11.6 (X); 8–16 (M)	24	75	USA
					11.8 (X); 8–16 (F)			
			School		12.9 (2.23); 8–17 (M)	374	48.6	
					13.17 (2.35); 8–17 (F)			
	March et al (1999)	57	School		13.98 (2.6); 8–18	142	35	USA
	Ross et al (2007)	58	Asthma		14.66 (1.75); 12–18	53	50.9	Canada
	Rynn et al (2006)	59	Anxiety disorder		12.2 (3.3); X	116	57.8	USA
			Depression		13.5 (3.1)	77	48.1	
	Thaler et al (2010)	60	Learning disability		13.75 (1.81); 11–17	41	78.6	USA
	White et al (2012)	61	HFASD		14.58 (1.67); 12–17	30	76.67	USA
	Wood et al (2002)	49	Outpatient anxiety clinic		11.71 (2.64); 8–17	186	53.8	USA
**RCADS**	Brown et al (2012)*	52	Pediatric primary care clinic sample		12.13 (2.7); 7–17	229	36.6	USA
	Chorpita et al (2000)	35	Schools		12.87 (2.82); 6.17–18.92	1641	45.6	Hawaii
	Chorpita et al (2005)	62	Clinical (CBT centre)		12.9 (2.7); 7.5–17.9	513	67.4	Hawaii
	Ebesutani et al (2010)	63	Clinical (CBT centre)		11.5 (2.5); 6.55–18.97	490	67.8	Hawaii
	Ebesutani et al (2011)	64	School		13.3 (2.98); 8–18	967	45.1	Hawaii
	Ebesutani et al (2012)	65	School		10.3 (1.7); 7–14	1060	39.3	Hawaii
			Clinic			303	67.3	
	Trent et al (2012)	66	School		Grades 2 to 12	12 659	49	USA
**RCMAS**	Cole et al (2000)	67	School		11.9 (0.5); 10.7–13.5	562 (T1)	49	USA
						630 (T2)		
	Dadds et al (1998)	68	School		X (X); 7–14	1786	40.9	Australia
	Dierker et al (2001)	53	School		14 (X); 13–15	632	45	USA
	Kenny & Faust (1997)	69	Mental health centre		10 (2.87); 5–16	54	64.9	USA
	Nelson & Renzenbrink (1995)	70	Psychiatric hospital		14.28 (1.57); 12–17	25	36	USA
	Olatunji & Cole (2009)	71	School		8.96 (0.61); 8–12	787	48	USA
	Paget & Reynolds (1984)	72	Learning disability		12.8 (1.2) 6–17	106	68.9	USA
	Perrin & Last (1992)	73	Clinic and community		X (X); 5–17	213	100	USA
	Pina et al (2009)	74	Anxiety clinic		10.21 (2.78); 6–16	677	52.7	USA
	Reynolds & Paget (1981)	75	School		X (X); 6–19	4972	50.2	USA
	Reynolds & Richmond (1997)	30	School		X (X); 6–18 (group 1)	329	X	USA
					X (X); 7–17 (group 2)	167		
	White & Farrell (2001)*	76	School		11.6 (0.6); 10.8–14.1	898	X	USA
	Wisniewski et al (1987)	77	School		12.1(0.92); 11–14	161	45	USA
**SCARED**	Bailey et al (2006)	78	Pediatric Primary care		14.54 (1.27); 8–17	190	51	USA
	Birmaher et al (1997)	31	Mood/anxiety disorders outpatient clinic		14.5 (2.3); 9–18	341	41	USA
	Birmaher et al (1999)	79	Mood/anxiety disorders clinic		13.8 (2.5); 9–19	190 C	48	USA
						166 P		
	Bodden et al (2009)	80	Clinically anxious		12.5 (2.7); 8–18	138	40	Netherlands
			Gen pop control		12.4(2.6) 8–18	38	37	
	Gonzalez et al (2012)**	81	Drawn from families seeking outpatient mental health services		13.96 (1.89); 11–18	374 C	53.9	USA
					10.69 (3.57); 5–18	808 P	62.3	
	Jastrowski et al (2012)	82	Pediatric chronic pain clinic		14.21 (2.54); 8–18	349	31	USA
	Monga et al (2000)	83	Mood/anxiety disorders outpatient clinic		14.4 (2.3); 9–18.9	295	43	USA
	Muris and Steerneman (2001)	84	Anxiety disorder or disruptive disorder		12.8 (2.4); 8–17	48	54.2	Netherlands
	Muris et al (1999)	85	School		12.2 (0.5); 11–14	101	47	Netherlands
					9.6 (1.1); 8–12	71	50.1	
					10 (1.2); 8–12	88	61.2	
	Muris et al (2004)	86	Anxiety outpatient		12 (2.9); 7–17	242	57.9	Netherlands
	Muris & Mayer et al (2001)	87	Clinically anxious		9.9 (1.4); 8–13	36	25	Netherlands
	Simon & Bogels (2009)	88	School	High anxious	9.92 (1.23); 8–13	188	45	Netherlands
				Median anxious	10.22 (1.13); 8–13	82	54	
	Van Steensel (2012)	89	ASD and anxiety disorder		11.37 (X); 7–18	115	78.3	Netherlands
			Anxiety disorder		12.79; 7–18	122	50.8	
	Wren et al (2007)	90	Pediatric Primary care		10.5 (1.4); 8–13	515	49.1	USA
**SCAS**	Brown-Jacobsen et al (2011)	40	Clinically anxious		12.32 (3.3);7–18	88	55.7	USA
	Essua et al (2011)***	91	General population		14.44 (1.7); 12–17	469	46.5	England
	Nauta et al (2004)	92	Clinically anxious; general population		10.8 (2.4); 6–18	745	52	Australia; Netherlands
	Russell & Sofronoff (2005)	93	AS; clinically anxious and normative data		X (X); 10–13	65 (AS)	84.6	Australia
	Spence (1998)	33	General population; clinical (separation and social phobia)		10.11 (1.25); 8–12	2052	42	Australia
	Spence (2003)	94	General population		13.51 (0.51); 13–14	875	54	Australia
	Whiteside & Brown (2008)	95	Community		12.74 (2.35); 9–18	85	55	USA
			Anxiety disorders		13.21 (2.81); 9–18	85	55	
	Whiteside et al (2012)	96	Community		10.42 (1.5); 8–13	420	49	USA
			Anxiety disorders		12.81 (3.1); 7–18	196	52	
**SWQ**	Bailey et al (2006)	78	Primary care		14.5 (1.27); 8–17	190	51	USA
	Russell & Sofronoff (2005)	93	AS; clinically anxious and normative data		X (X); 10–13	65 (AS)	84.6	Australia
**CGI**	Lewin et al (2012)	97	OCD		12.2 (2.5); X	71	36.6	USA

ADIS: Anxiety Disorders Interview Schedule. MASC: Manifest Anxiety Scale for Children. RCADS: Revised Child Anxiety and Depression Scale. RCMAS: Revised Child Manifest Anxiety Scale. SCARED: Screen for Child Anxiety and Related Emotional Disorders. SCAS: Spence Children's Anxiety Scale. SWQ: Social Worries Questionnaire. CGI: Clinical Global Impressions.

X =  No details. M =  male; F =  female. C: child; P: parent. AS: Asperger Syndrome. ASD: autism spectrum disorder. CBT: cognitive behaviour therapy. HFASD: high-functioning ASD. OCD: obsessive-compulsive disorder. T1 =  Time 1; T2 =  Time 2.

looked at cross ethnic validity (all participants African American); ** looked at cross ethnic validity (all participants African American or non Hispanic white); *** Data collected from 5 European countries – details are for UK sample.

Only four articles assessing measurement properties included an ASD sample, reporting on use of five of the tools (i.e. not RCADS, RCMAS or CGI). The majority of the studies were carried out in the USA. The methodological quality of each article is presented in Table 4. None of the articles had looked at measurement error, so this property is not included in the table. Only one article reported responsiveness to change. The synthesised evidence on the quality of the measurement properties of the individual tools is shown in Table 5. To aid interpretability [39], it is important to have evidence on differences in scores between subgroups (including normative data) and this was available in many of the articles; however, no article reported on levels of minimal important change, nor on floor and ceiling effects.

**Table 4:** Methodological quality of each article per measurement property and instrument according to COSMIN Checklist.

Measure	Article	Ref No.		Version	Internal Consistency	Reliability	Content Validity	Structural Validity	Hypothesis Testing	Criterion Validity	Responsiveness
**ADIS**	Brown-Jacobsen et al (2011)	40		C + P	-	-	-	-	Good	-	-
	Canavera et al (2009)	41		C + P	-	-	-	-	Good	-	-
	Comer & Kendal (2004)	42		C + P	-	Good	-	-	Good	-	-
	Grills & Ollendick (2003)	43		C + P	-	Good	-	-	Good	-	-
	Higa-McMillan (2008)	44		C	-	-	-	good	-	-	-
	Lyneham et al (2007)	45		C + P	-	good	-	-	-	-	-
	Lyneham & Rapee (2005)	46		C + P	-	good	-	-	-	-	-
	Silverman et al (2001)	47		C + P	-	good	-	-	good	-	-
	Storch et al (2012)	48		C + P	-	-	-	-	good	-	-
	Wood et al (2002)	49		C + P	-	-	-	-	good	-	-
**MASC**	Anderson et al (2009)*	50		C	good	-	-	-	excellent	excellent	-
	Baldwin & Dadds (2007)	51		C + P	excellent	-	-	excellent	good	-	-
	Brown et al (2012)	52		C	good	-	-	good	-	-	-
	Dierker et al (2001)	53		C	fair	-	-	-	-	good	-
	Grills-Taquechel et al (2008)	54		C	excellent	-	-	good	-	excellent	-
	Langer et al (2010)	55		C + P	fair	-	-	-	-	good	-
	March et al (1997)	56		C + P	excellent	poor α	-	excellent	poor α	-	-
	March & Sullivan (1999)	57		C	-	good	-	-	-	-	-
	Ross et al (2007)	58		C	-	-	-	-	-	good	-
	Rynn et al (2006)	59		C	good	-	-	good	good	excellent	-
	Thaler et al (2010)	60		C + P	fair	-	-	-	fair	fair	-
	White et al (2012)	61		C + P	fair	-	-	-	fair	-	-
	Wood et al (2002)	49		C + P	-	-	-	-	-	excellent	-
**RCADS**	Brown et al (2012)	52		C	good	-	-	good	-	-	-
	Chorpita et al (2000)	35		C	excellent	good	good	excellent	good	-	-
	Chorpita et al (2005)	62		C	excellent	-	-	excellent	fair	excellent	-
	Ebesutani et al (2010)	63		P	excellent	-	-	excellent	good	excellent	-
	Ebesutani et al (2011)	64		P	excellent	good	-	excellent	excellent	-	-
	Ebesutani et al (2012)	65		C Short	excellent	-	-	excellent	-	excellent	-
	Trent et al (2012)	66		C	excellent	-	-	excellent	-	-	-
**RCMAS**	Cole et al (2000)**		67	C + P	poor β	-	-	excellent	-	-	-
	Dadds et al (1998)***		68	C	good	-	-	-	good	-	-
	Dierker et al (2001)		53	C	fair	-	-	-	good	good	-
	Kenny & Faust (1997)		69	C	-	-	-	-	good	-	-
	Nelson & Renzenbrink (1995)		70	C	-	poor	-	-	poor α	-	-
	Olatunji & Cole** (2009)		71	C	good	excellent	-	excellent	-	-	-
	Paget & Reynolds (1984)		72	C	poor β	-	-	poor α	-	-	-
	Perrin & Last (1992)		73	C	-	-	-	-	-	fair	-
	Pina et al (2009)		74	C	-	-	-	excellent	fair	-	-
	Reynolds & Paget (1981)		75	C	-	-	-	excellent	-	-	-
	Reynolds & Richmond (1997)		30	C	poor	-	fair	-	fair	-	-
	White & Farrell (2001)		76	C	-	-	excellent	excellent	-	-	-
	Wisniewski et al (1987)		77	C	-	good	-	-	-	-	-
**SCARED**	Bailey et al (2006)*		78	C + P	-	-	-	-	-	excellent	-
	Birmaher et al (1997)		31	C + P	excellent	fair	good	good	fair	excellent	-
	Birmaher et al (1999)		79	C + P	poor α	-	-	poor α	fair	fair	-
	Bodden et al (2009)		80	C + P SCARED-71	good	-	-	-	-	excellent	-
	Gonzalez et al (2012)		81	C + P	good	-	-	excellent	-	excellent (P)	-
	Jastrowski et al (2012)		82	C + P	excellent	-	-	excellent	good	-	-
	Monga et al (2000)		83	C + P	-	-	-	-	excellent	excellent	-
	Muris et al (1999)		85	C + P SCARED-R	good	good	-	-	good	-	-
	Muris et al (2004)		86	C + P SCARED-R	poor	-	-	-	fair	excellent	-
	Muris & Mayer et al (2001)		87	C SCARED-R	-	-	-	-	-	fair	fair
	Muris and Steerneman (2001)		84	C SCARED-R	fair	-	-	-	fair	fair	-
	Simon & Bogels (2009)		88	C SCARED-71	-	-	-	-	-	excellent	-
	Van Steensel (2012)		89	C + P SCARED-71	good	-	-	-	good	excellent	-
	Wren et al (2007)		90	C + P	-	-	-	excellent	good	-	-
**SCAS**	Brown-Jacobsen et al (2011)		40	C + P	good	-	-	-	good	good	-
	Essua (2011)		91	C	excellent	-	-	excellent	good	-	-
	Nauta (2004)		92	P	excellent	-	-	excellent	good	excellent	-
	Russell & Sofronoff (2005)		93	C + P	-	-	-	-	good	-	-
	Spence (1998)		33	C	excellent	good	-	excellent	good	excellent	-
	Spence et al (2003)		94	C	excellent	good	-	excellent	good	-	-
	Whiteside & Brown (2008)		95	C + P	good	-	-	-	good	good	-
	Whiteside et al (2012)****		96	C + P	good	-	-	-	good	good	-
**SWQ**	Bailey et al (2006)		78	P	-	-	-	-	-	excellent	-
	Russell & Sofronoff (2005)		93	C + P	-	-	-	-	good	-	-
**CGI**	Lewin et al (2012)		97	Improvement	-	good	-	-	-	-	-

ADIS: Anxiety Disorders Interview Schedule. MASC: Manifest Anxiety Scale for Children. RCADS: Revised Child Anxiety and Depression Scale. RCMAS: Revised Child Manifest Anxiety Scale. SCARED: Screen for Child Anxiety and Related Emotional Disorders. SCAS: Spence Children's Anxiety Scale. SWQ: Social Worries Questionnaire. CGI: Clinical Global Impressions.

Measurement Error was not evaluated in any article; Cross cultural validity was not included in the review..

C =  child self report; P =  parent report. R =  Revised.

looked at particular subscale (Social Anxiety/Social Phobia); ** created continuous data by altering response format; *** looked at particular subscale (total score and lie); **** looked at particular subscale (OCD).

α small sample; β no alpha for subscales.

**Table 5:** Quality of measurement properties of tools.

		Measurement properties								Interpretability
Tool	Version	Internal Consistency	Reliability	Content Validity	Structural Validity	Hypothesis Testing		Criterion Validity	Responsiveness	Differences in scores between subgroups
						Par/ch **	Conv/div ***			
**Anxiety Disorders Interview Schedule (ADIS)**	C	na	+++ *	na	++	− − −	na	Na	na	na
	P	na	+++ *	na	na	− − −	na	Na	na	na
**Multidimensional Anxiety Scale for Children (MASC)**	C	+++	++	na	+/−	− − −	+++	+/−	na	Y
	P	+++	++	na	+/−	− − −	++	+/−	na	Y
**Revised Children**'**s Anxiety and Depression Scale (RCADS)**	C	+++	− −	++	+/−	na	++	+++	na	Y
	P	+++	++	na	− − −	na	+++	+++	na	Y
	C Short	+++	Na	na	+++	na	na	+++	na	Y
**Revised Children**'**s Manifest Anxiety Scale (RCMAS)**	C	++	+/−	+++	+++	− −	+/−	+/−	na	Y
	P	?	Na	na	?	na	na	Na	na	Y
**Screen for Child Anxiety Related Emotional Disorders (SCARED)**	C	+++	+	++	+/−	− −	na	+++	na	Y
	P	+++	+	++	+/−	− −	na	+++	na	Y
	C – Rev	++	++	na	na	+/−	++	++	+	Y
	P – Rev	++	na	na	na	+/−	?	++	na	Y
	C – 71	+++	na	na	na	++	++	+/−	na	Y
	P – 71	+++	na	na	na	++	− −	+/−	na	Y
**Social Worries Questionnaire (SWQ)**	C	na	na	na	na	− −	na	na	na	Y
	P	na	na	na	na	− −	na	++	na	Y
**Spence Children**'**s Anxiety Scale (SCAS)**	C	+++	− −	na	+++	+++	+++	+++	na	Y
	P	+++	− −	na	++	+++	+++	+++	na	Y
**Clinical Global Impressions (CGI)**	CGI-I	na	+	na	na	na	na	na	na	na

C =  child self report; P =  parent report. Rev =  Revised.

3 studies of inter-rater, 1 study of test-retest reliability for both ADIS-C and ADIS-P; 1 study of face-to-face and telephone agreement for ADIS-P.

parent-child agreement.

convergent/divergent validity: correlations ≥0.50 with other scales measuring the same construct, and higher than with unrelated constructs.

na =  no information available.

The ADIS is a clinical interview, with entry-level questions which determine which areas of anxiety disorder are explored. The recommended procedure is that parent and child are interviewed separately, and then the interviewer determines the disorder diagnoses and clinical severity rating. When the separate interviews are compared, agreement is low both at the level of whether a disorder is indicated and at symptom level (though one study [42] found the latter to be higher). As a clinical interview, some measurement properties such as internal consistency and content validity have not been studied, with the latter presumably assumed because the measure was developed from the Diagnostic and Statistical Manual of Mental Disorders [17]. In many studies ADIS is used as the ‘gold standard’ against which questionnaire measures are compared. Its strengths lie in inter-rater reliability, and evidence also of test-retest reliability.

Turning to the questionnaire measures, evidence for internal consistency of the parent and child versions of the SCAS was strong for total and subscale scores, apart from the fear of physical injuries subscale [33]; [40]; [94]; [95] and generalised anxiety disorder (GAD) subscale [40]. Test-retest reliability for child report was r = .60 [33] at 6 months, and r = .63 at 3 months [94] which seems acceptable (the COSMIN criterion of r≥0.80 may be set unduly high for a subjective measure of feelings).

Evidence for the structural validity of the six factor structure for the SCAS child version was strong [33]; [91]; [94], though lower for the parent version the confirmatory factor analysis finding only acceptable evidence of fit [98] (root mean square error of approximation (RMSEA)  = .075) [92]. Criterion validity of the SCAS was supported by significantly higher scores in a clinical than a non-clinical group [33]; [92]; [95], more than 80% of those with an anxiety disorder correctly classified, and discrimination between disorders good apart from GAD and panic-agoraphobia [92]. Convergent and divergent validity were demonstrated by significantly higher correlations between the child report SCAS and RCMAS than with the Child Depression Inventory (CDI) [33]; [94]; and furthermore by significantly higher correlations between SCAS parent and the Child Behavior Checklist (CBCL) internalising than externalising scales [92], and higher between SCAS child and the Strengths and Difficulties Questionnaire emotional subscale than with the conduct or hyperactivity subscales [91].

Findings for parent-child agreement for the SCAS depended on the analysis conducted. Using ANOVA, it was found that parents rated significantly higher than children on all subscales apart from OCD and panic-agoraphobia [93]. In contrast, studies reporting correlations [40]; [95]; [96] consistently found r>.50 on total and subscale scores, apart from on GAD [40].

The RCADS was developed as a revision of the SCAS, in order to correspond to dimensions of several DSM-IV anxiety disorders and also to include major depression. In particular, it was intended to refine the measurement of GAD to reflect core aspects of ‘worry’. Internal consistency was found to be good for subscales, and also for the shortened Anxiety 15 item version. In the original study [35] one week test-retest reliability ranged from r = .65 to .80. The total variance explained by the factor analysis was less than 50%; however, subsequent confirmatory factor analyses have reported good fit to the 6 factor solution [52]; [62]; [66] for the child scale, and acceptable for the parent scale [63]; [64]. Convergent and divergent validity have been shown convincingly, as has criterion validity with diagnoses based on standardised clinical psychiatric interview.

The MASC has well-established strengths in internal consistency (except for the subscale Harm Avoidance in [55]) and in test-retest reliability. The latter has been shown at 3 weeks [56]; [57] and 3 months [56]; indeed [60] have shown stability estimates around r = 0.50 for child-report and higher (.56 to .70) for parent-report in a community sample. The original factor analysis [56] for the MASC explained only 39.4% of the total variance; however, subsequent confirmatory factor analyses have found good fit for the four factor solution [51]; [52]; [54] though one study [59] found only acceptable evidence of fit (e.g. RMSEA  = 0.73) [98]. Correlations with other measures of anxiety are high, with discriminant validity established (usually by lower agreement with scales measuring depression). Findings for criterion validity have been variable, showing high levels of agreement with diagnostic groupings (diagnostic interview or ADIS as ‘gold standard’) in some community [50]; [54] and clinical studies [59;60;49– except for generalised anxiety disorder], but not in other clinical studies [55]; [58] and in the school-based study by Dierker and colleagues [53] where generalised anxiety disorder was well predicted in girls, but social phobia and specific phobia were not. As for ADIS and SCARED (below), agreement between child and parent report was low [51]; [60]; [61]; In the MASC source paper [56] mother-child agreement was only r = .39, and father-child and father-mother agreement were negligible.

Articles generally report high internal consistency of the RCMAS but often do not give figures for the subscales. Only one study reported test-retest reliability, which was high (one week r = .88; five week r = .77). One study hypothesised stability of scores for psychiatric inpatients over a 4 week period, but instead found reduction in anxiety not substantiated by clinical rating [70]. Both content validity and structural validity appear strong. The latter has been examined in a number of ways, with several studies considering congruence of factors and their relationships across parent/child or different ethnic groups. However, one small study of children with learning disability [72] reported a lower proportion of variance accounted for by the general anxiety factor than was found in the normative sample. Some RCMAS articles suggested convergent and divergent validity, but the better quality studies found less convincing results. The one study to compare RCMAS child report with parent (parents completed the Revised Behavior Problem Checklist) found significant disagreement [69]. The two studies of criterion validity against diagnostic interview produced conflicting results; the clinic study supported criterion validity [73] but the community study concluded that the RCMAS was less successful than the MASC in identifying anxiety and depression [53].

There are a number of versions of the SCARED. The original 38 and 41 item tools have good content validity being derived from DSM [31], some evidence of test-retest reliability for total and subscale scores on both parent and child versions [31], plus consistently good internal reliability. Good structural validity was found [82] though evidence for measurement invariance was not as strong (RMSEA >.06) [81]. Criterion validity was good [78]; [81]: clinically anxious children scored significantly higher on the child SCARED than non-anxious, depressed and disruptive groups on total and subscale scores [79]; [83], and by examining area under the curve (AUC) against clinical interview [78]; [83].

The SCARED-Revised is a 66 item measure with nine subscales. Internal consistency was found to be good though the quality of the articles varied. The total scores and most of the subscales had good internal consistency, except OCD (parent and child versions), blood/injection/injury (child) and environmental/situational (parent) [85] and specific phobias [84]. Test-retest reliability of the child total score was positive (r>.80) with the subscales approaching this level apart from GAD, separation, OCD and traumatic stress (r<.70) [85]. Correlations across time with the State Trait Anxiety Inventory for Children demonstrated responsiveness to change though the quality of the evidence was limited [87]. Significantly higher SCARED-R scores were predictive of those with anxiety disorders, demonstrating criterion validity, though the GAD, specific phobias and separation anxiety subscales performed less well in the child version [86]. Correlations between parent and child were mixed with both high [86] and low [85] agreement found.

The SCARED-71 is a version adding five further social phobia items to the SCARED-R. Internal consistency was positive in parent and child versions for total and all but one subscale scores (OCD, child report) [89]. Criterion validity in terms of predictability of diagnosis by corresponding subscale was good except for GAD [88]. However, correlations with ADIS parent report were low, for both anxiety disorder and ASD groups [89].

The parent version of the Social Worries Questionnaire has good evidence of criterion validity with agreement for social phobia (AUC >.80) as measured by the ADIS [78]. Parent reports on the SWQ also demonstrated that children with Asperger syndrome were significantly more anxious than typically developing children, on a par with a clinically anxious sample. As predicted by Russell and Sofronoff, parent and child reports of anxiety differed [93].

The CGI-I showed inter-rater agreement for parent-child, therapist-parent, therapist-child, and independent evaluator-parent though most of the correlations were <.70. Across time, improvement was reported significantly sooner by parents and children than by therapists and the independent evaluator, though judgements tended to converge by 14 weeks of treatment for OCD.

## Discussion

### Principal Findings

In this systematic review, eight tools were found which had been used to measure primary outcomes in anxiety intervention trials for children with high-functioning ASD in middle childhood. A second systematic search of literature found sixty-three articles studying children and examining the measurement properties of the eight tools.

There was limited or no evidence for three of the eight properties of measurement tools rated in this review using the COSMIN checklist: measurement error, content validity and responsiveness to change. In terms of the primary purpose of the review – to inform the choice of tool to measure outcomes of intervention trials for anxiety in children with ASD – these are serious limitations in the evidence.

Only four articles included children with ASD, and none of these considered content validity. Indeed, the field is hampered by lack of a definitive conceptualisation of anxiety in ASD, and the means to capture features of anxiety as a clinical disorder separate from ASD [15]; [99]–[102]. Anxiety interacts with core symptoms (such as poor social skills and repetitive thoughts) and so differs in several ways from anxiety seen in typically developing children. For example, a child with ASD who is reluctant to go to school is more likely to be experiencing social anxiety rather than separation anxiety. However, until basic psychometric work including content analysis is carried out, outcome measures developed with typically developing children will continue to be utilised with children with ASD [100]; [101].

The lack of evidence about responsiveness to change of tools is also a limitation for the purpose of the review. The CGI Improvement rating explicitly focuses on change, and was utilised by three of the ASD intervention studies, indicating treatment effects. It has been used widely in autism medication trials [6], has comparable effect sizes to other rating scales in adult anxiety intervention trials [103], and has the advantage that it can be rated blind to group and time point. Therefore it is likely to continue to be used in intervention trials for children, even though evidence for its measurement properties was sparse in this review.

One further property included in the COSMIN checklist was not included in the review, cross-cultural validity. However support for the measurement properties of the SCARED across several countries and cultures has been found in a meta analysis [104] and by Gonzalez and colleagues [81].

Overall the findings of the review suggest that the tools which are most robust in their measurement properties are the Spence Children's Anxiety Scale, its revised version – the Revised Children's Anxiety and Depression Scale, and also the Screen for Child Anxiety Related Emotional Disorders. The weakness of the measurement of GAD by the SCAS appears to have been improved in the RCADS. However, self-report and parent report generally have the limitation in RCTs of therapy that they are not ‘blinded’. In the four ASD intervention trials which used ADIS, participants were asked not to unblind the researcher as they described current events and behaviours in the clinical interview. Thus a combination of ways of measuring anxiety (feelings and behaviours) appears to be necessary to achieve robust measurement.

### Clinical Implications

The review found a mixed picture in terms of the level of correlation between parent and child report. Agreement is not necessarily to be expected, with each individual reflecting different symptoms captured (for example, more observable behaviours being identified by the parent), and the possible influence of factors such as the parent's own experiences affecting sensitivity to the child's symptoms [105]. While the level of agreement between parents and their children with ASD may actually be higher than observed for typically developing groups [106], a number of researchers [e.g. 26;61;93] comment that children with high functioning ASD are likely to under-report anxiety symptoms, one reason being difficulty in identifying their own (and others') emotions. Therefore a combination of perspectives is likely to give a more rounded picture.

One further issue for the measurement of outcomes in intervention trials and clinical practice in ASD is a need to consider further what constitutes a successful outcome [13]. Necessarily, the tools reviewed here as primary outcomes focus on clinical symptoms; however, the goals of intervention are likely to include broader constructs such as participation and quality of life. The International Classification of Functioning, Disability and Health paradigm [107], which is the World Health Organisation recommended conceptual model for measuring health and disability and evaluating interventions, emphasizes that body functions, activity and participation may all be important indicators of intervention success. Children with ASD may have hypersensitivity to visual and auditory stimuli, which in turn may result in activity limitations (e.g. social anxiety) and restricted social participation (e.g. reluctance to go to new places). Effective interventions for anxiety would also expect to see change in socially valid outcomes for children such as new experiences and greater success in friendships, whatever the nature of the baseline anxiety.

### Limitations

This systematic review had some limitations. Articles were accessed only in English as we lacked resources for translation. Data extraction was done only in part by two independent reviewers. Although the COSMIN manual and checklist is validated and well structured, there is still an element of subjectivity in the review process such that different decisions regarding ratings and synthesis might be made by other reviewers.

The focus was on children in middle childhood who are high-functioning, and anxiety measurement issues in other age and ability groups have not been considered. Nevertheless, children with ASD are reported to be vulnerable to high anxiety across ages [8]; [108] and abilities [8]; [108]; [109] and so intervention and measurement issues require wider examination.

## Conclusions

Though there appears to be a certain international practice consensus developing in research groups undertaking trials of intervention for anxiety in children with ASD, the evidence for the measurement properties of the chosen tools is patchy. The review has allowed some conclusions to be drawn on what may be the psychometrically sound assessment tools. However, there requires to be further consideration of how to achieve blinded outcome measurement in RCTs, and how to judge the appropriateness of tools developed to measure anxiety in typically developing children when applied with children who have ASD.

## Supporting Information

Checklist S1 **PRISMA checklist.**
(DOC)
